# The Role of Glutamine in the Complex Interaction between Gut Microbiota and Health: A Narrative Review

**DOI:** 10.3390/ijms20205232

**Published:** 2019-10-22

**Authors:** Simone Perna, Tariq A. Alalwan, Zahraa Alaali, Tahera Alnashaba, Clara Gasparri, Vittoria Infantino, Layla Hammad, Antonella Riva, Giovanna Petrangolini, Pietro Allegrini, Mariangela Rondanelli

**Affiliations:** 1 Department of Biology, College of Science, University of Bahrain, 32038 Sakhir, Bahrain; talalwan@uob.edu.bh (T.A.A.); zahraaj.alaali@gmail.com (Z.A.); tahera.alnashaba@gmail.com (T.A.); Laylabh2018@outlook.com (L.H.); 2 Endocrinology and Nutrition Unit, Azienda di Servizi alla Persona ‘‘Istituto Santa Margherita’’, University of Pavia, Pavia 27100, Italy; gasparri.clara@gmail.com; 3 Department of Biomedical Science and Human Oncology, University of Bari, Bari 70121, Italy; viriainfantino@hotmail.it; 4 Research and Development Department, Indena SpA, 20139 Milan, Italy; 5 IRCCS Mondino Foundation, Pavia 27100, Italy; mariangela.rondanelli@unipv.it; 6 Department of Public Health, Experimental and Forensic Medicine, Unit of Human and Clinical Nutrition, University of Pavia, Pavia 27100, Italy

**Keywords:** glutamine, glutamate, obesity, amino acids, gut, microbiome, microbiota, diet

## Abstract

The scientific literature has demonstrated that glutamine is one of the main beneficial amino acids. It plays an important role in gut microbiota and immunity. This paper provides a critical overview of experimental studies (in vitro, in vivo, and clinical) investigating the efficacy of glutamine and its effect on gut microbiota. As a result of this review, we have summarized that glutamine could affect gut microbiota via different mechanisms including the reduction in the ratio of *Firmicutes* to *Bacteroidetes,* with the activation of NF-κB and PI3K-Akt pathways, reducing the intestinal colonization (*Eimeria* lesions) and bacterial overgrowth or bacterial translocation, increasing the production of secretory immunoglobulin A (SIgA) and immunoglobulin A+ (IgA^+^) cells in the intestinal lumen, and decreasing asparagine levels. The potential applications of glutamine on gut microbiota include, but are not limited to, the management of obesity, bacterial translocation and community, cytokines profiles, and the management of side effects during post-chemotherapy and constipation periods. Further studies and reviews are needed regarding the effects of glutamine supplementation on other conditions in humans.

## 1. Introduction

Glutamine is the most abundant free amino acid in the human body that is utilized by the intestinal endothelium. The importance of glutamine in intestinal physiology and management of several gastrointestinal diseases has been reported before [1]. Glutamine has been shown to promote enterocyte proliferation, regulate tight junction proteins, suppress proinflammatory signaling pathways, and confer protection against apoptosis and cellular stresses during normal and pathological conditions [2].

The importance of glutamate in nitrogen metabolism in enteric bacteria such as *Bacteroides thetaiotaomicron* is well documented, and it has been hypothesized that plasma and fecal levels of glutamate are influenced by the composition of the gut microbiota [3]. Moreover, a healthy gut microbiota is characterized by its plasticity, that is, the amount of daily variability in an individual’s microbiota composition in response to dietary habits [4].

Some functional amino acids, such as tryptophan and glutamine, have beneficial effects on the gut-associated immune system through the modulation of key metabolic signaling pathways in the intestinal barrier, often involving crosstalk with their receptors or ligands [5]. However, more studies on the intestinal microbiota are needed to broaden our understanding of underlying mechanisms of dietary amino acids in metabolic processes in humans and animals.

This review highlights the amino acid glutamine-mediated crosstalk between intestinal immunity and microbiota, with an emphasis of the role of glutamine in regulating the microbiome–health axis.

## 2. Overview of Glutamine Mechanisms

Glutamine has been known for its beneficial effect on different organs and body systems. It is associated with many mechanisms and metabolic pathways including reactions catalyzed by glutaminase, aspartate aminotransferase, oxoglutarate dehydrogenase, succinate dehydrogenase, fumarase, malate dehydrogenase, and phosphoenolpyruvate carboxykinase. The mechanisms of the potentially protective effects of glutamine have been investigated and include maintenance of intestinal mucosal integrity, modulation of inflammatory response, nucleotide biosynthesis, energy metabolism, and stimulation of immunity [6,7,8,9].

Xue et al. [10] using a rat model reported several protective mechanisms of glutamine including increased proportion of reduced glutathione, heat-shock protein induction, and increased proportions of cytotoxic T (CD3^+^CD8^+^) and memory CD8^+^ cells in mesenteric lymph nodes. Glutamine also prevents the CPT-11-induced increase of *β*-glucuronidase activity in the cecum, suggesting that glutamine affected intestinal microbiota [10]. For this reasons, glutamine has been used to enhance gut mucosal repair in models of chemotherapy, sepsis, trauma, and inflammation [11].

Furthermore, glutamine can activate ornithine descarboxylase, a key enzyme involved in polyamine synthesis, thereby enhancing DNA synthesis of intestinal epithelial cells [12]. Additionally, glutamine can activate mitotic signaling pathways, including nitrogen-activated protein kinase and transcription factors, leading to proliferative responses [13,14].

Oral glutamine administration was demonstrated to reduce the occurrence of bacterial translocation in a rat model by inhibiting bacterial endocytosis by enterocytes or adhesion to the enteric wall in the presence of bile [15]. Similarly, Azuma et al. [16] reported that a commercial enteral supplementation product enriched with glutamine, dietary fiber, and oligosaccharide (GFO) prevented gut bacterial translocation in an experimental mouse infection model. Glutamine supplementation was also reported to ameliorate constipation and improve intestinal function by regulating endogenous gut microbiota [17] and increasing the nitrogen and energy harvesting capacity [18]. For this reason, glutamine is currently considered as a potential therapeutic option for the treatment of constipation.

## 3. Results

### 3.1. Glutamine and Immune Cells

Several studies were undertaken to determine the relationship between glutamine and immune cell function. A study by Newsholme et al. [7] was the first to demonstrate that glutamine is essential for lymphocyte proliferation and production of cytokines. In particular, the utilization of glutamine by cells of the immune system such as lymphocytes, neutrophils, and macrophages is fourfold greater than that of glucose as indicated by the production of glutamate, aspartate, lactate, and ammonia [7]. Similarly, a study of Ardawi et al. [8] resulted in lower rates in the absence of glutamine in incubated lymphocytes isolated from rat mesenteric lymph nodes. Additionally, in another study by Ardawi [8], glutamine was metabolized at a higher rate in vitro by resting and mitogen-stimulated human peripheral lymphocytes. Nevertheless, it is worth noting that the glutamine metabolism pathway includes reactions catalyzed by glutaminase, aspartate aminotransferase, oxoglutarate dehydrogenase, succinate dehydrogenase, fumarase, malate dehydrogenase, and phosphoenolpyruvate carboxykinase [7]. Furthermore, glutamine is involved in a number of key functions in the immune system, such as being a precursor for ornithine synthesis in macrophages and monocytes [9]. This is in line with a study by Calder et al. [19] who demonstrated that macrophage-mediated phagocytosis is influenced by glutamine availability. Moreover, the authors also noted that glutamine may play a role as a precursor for glutathione synthesis and as such may play a direct role in antioxidant defenses in these cells. In addition, glutamine may act as an anaplerotic substrate in the ß-cell, via formation of glutamate and 2-oxoglutarate [9].

Nevertheless, many studies [8,9,10,11] have shown that glutamine levels in plasma and skeletal muscle are lowered under catabolic conditions, such as sepsis, injury, burns, or surgery, as well as physical activity and endurance exercise. Decreased glutamine plasma levels may be the result of increased demand for glutamine by the immune system and other tissues, such as the liver, kidney and gut. It has been suggested that the lowered plasma glutamine concentration contributes, at least in part, to the immunosuppression which accompanies such situations [19].

### 3.2. Glutamine Supplementation and Gene Expression

Glutamine undergoes a partial oxidation process called glutaminolysis. This process serves an important role in providing nitrogen and carbon for precursors for synthesis of macromolecules essential for cell proliferation and tissue regeneration, such as nucleic acids (DNA and RNA) [13]. High rates of glutaminolysis also provide an ideal condition for the precise and sensitive control of the use rate of the intermediates for biosynthesis when required without any need for extracellular signals [7]. Furthermore, an in vivo study on hamsters demonstrated that glutamine can activate ornithine descarboxylase, a first and rate-limiting enzyme in polyamine synthesis in a dose- and time-dependent approach, thereby enhancing DNA synthesis [14].

Dietary glutamine supplementation has been demonstrated to attenuate oxidative stress-related gene expression, enhance the antioxidant capacity, and suppress renal thioredoxin-interacting protein expression in rats with streptozotocin-induced diabetes [20]. In another study, glutamine supplementation was reported to partially prevent the increase in p38 mitogen-activated protein kinase (p38) and c-Jun *N*-terminal kinase (JNK) phosphorylation in neutrophils obtained from rats, thereby reducing exercise-induced apoptosis [21].

### 3.3. Effect of Glutamine on Enterocytes

Many studies suggested that enterocytes are responsible for a great deal of glutamine production and metabolism in isolated preparations of the intestinal mucosa [22,23,24]. To specify, glutamine metabolism proceeds by two steps. First, glutaminase catalyzes the conversion of glutamine to glutamate. The following step is the transamination of glutamate via aspartate aminotransferase and alanine aminotransferase to produce α-ketoglutarate, alanine, and aspartate. The end products of glutamine metabolism by incubated gut preparations in vitro (mainly alanine) suggest that enterocytes are responsible for most gut glutamine metabolism [25]. Indeed, utilization of glutamine by isolated human enterocytes was previously reported to be at about 14.90 μmol/min/g dry cell weight [23]. Another study has demonstrated such utilization to be associated with a major increase in oxygen consumption following prolonged exercise [24].

Moreover, provision of exogenous glutamine has had beneficial effects on humans and animals, particularly in improving intestinal function. For example, parenteral glutamine supplementation results in an improvement in nitrogen balance, gut barrier function, and diminished incidence of infection in critically ill patients or patients after surgery [26].

### 3.4. Clinical Application of Glutamine Supplementation

Glutamine supplementation is used in several clinical applications in patients with trauma, burns, and following injuries. During severe metabolic stress, more glutamine must be produced and released to meet the increased metabolic demands for rapidly dividing cells, such as in the gut and immune system [27]. Glutamine depletion may contribute to infections, weight loss, and muscle-wasting in trauma and critically ill patients [28]. Such conditions have been proposed as indications for glutamine supplementation.

Numerous randomized clinical trials and experimental studies demonstrated that glutamine supplementation has a beneficial effect on different organs and systems in the body [9,26,29]. In addition, there is evidence that parentally administrated glutamine does not appear to pose a health risk, or cause detrimental changes to the central nervous system and alterations in blood chemistry [22]. The authors did, however, report a higher content of plasma glutamine within five days following treatment with glutamine [22]. Similarly, no adverse effects were reported when patients undergoing elective abdominal surgery were administered with 0.285 g/kg body weight of glutamine-enriched total parenteral nutrition [22]. In fact, patients receiving the glutamine formula exhibited an improvement in nitrogen balance and a sparing of the intramuscular glutamine pool [22]. Furthermore, evidence points towards enteral feeding still being the method of choice and use of increasing the concentration of glutamine in the systemic circulation. Indeed, studies on healthy adults suggest that 40–50% of radioactively labelled glutamine enters the circulating pool, while the remaining 50–60% is consumed by the splanchnic bed [13,22,29].

### 3.5. Glutamine Supplements on the Gut Microbiota of Obese and Overweight Individuals

As demonstrated by the work of de Souza et al. [30], glutamine supplementation affects the gut microbiota composition of obese individuals. The authors of that study found that glutamine supplementation decreased the *Firmicutes*-to-*Bacteroidetes* ratio (from 0.85 to 0.57) and reduced *Actinobacteria* in obese individuals, compared to alanine supplementation. Further evidence connecting glutamate to gut microbiota composition was recently presented by Palomo-Buitrago et al. [31], who showed that obese subjects tend to have a lower fecal glutamate and glutamate/glutamine ratio than non-obese individuals. Moreover, they concluded that *Coriobacteriaceae*/*Streptococaceae* and *Coribacteriaceae*/*Strepttococaeae* ratios were associated both with scores from the neuropsychological Trail Making Test Part A (TMT-A) and fecal glutamate/glutamine ratios [31].

### 3.6. Effect of Glutamine on Gut Microbiota during Post-Chemotherapy

Previous studies have documented a range of gastrointestinal bacterial infections associated with various chemotherapeutic agents [32,33,34]. Only one study was carried out on a colon cancer-bearing rat model, showing that glutamine treatment prevented the reduced abundance of β-glucuronidase-positive bacterial species (*Clostridium* cluster XI and *Enterobacteriaceae*) following the administration of the chemotherapeutic agent irinotecan [35]. The authors suggested that glutamine supplementation may have a role in ameliorating chemotherapy-induced increase of β-glucuronidase activity in the cecum [35].

### 3.7. Effect of Glutamine on the Microbiota in Different Types of Diet

Several studies were carried out in vivo in animal models to determine the influence of dietary supplementation with glutamine on the intestinal microbiota composition. For instance, one study reported no effect of glutamine supplementation on *Clostridium* Cluster I/II levels, which remained undetectable, as well as on restoring total fecal bacteria levels or fecal *Bifidobacterium* and *Lactobacillus* groups in fructose-fed rats [36]. Similarly, a study by Chamorro et al. [37] on growing rabbits showed no effect of diets supplemented with glutamine on the growth rates in fatteners. The authors did, however, report that glutamine supplementation (from 0.5% to 1% of diet) reduced the presence of *Clostridium* spp. from 86.7% to 33.3% (*p*-value = 0.003) in the ileum and *Helicobacter* spp. and decreased from 86.7% to 46.7% (*p*-value = 0.003) and from 86.7% to 46.7% (*p*-value = 0.028) in the ileum and cecum, respectively [37].

On the other hand, a more recent study showed a positive correlation between glutamine supplementation in cultured turbot and weight gain, feed efficient ratio, villous height and crypt depth, integrity of the microvilli, serum lysozyme activity, and serum concentrations of complement (C3, C4) and immunoglobulin (IgM) [38]. A similar trend was also shown by a previous study that investigated the effect of dietary glutamine on growth performance, innate immune responses, and intestinal structure of juvenile red drum fish [39]. The authors concluded that glutamine supplementation may enhance the feed efficiency of red drum as well as promoting the development of the intestine [39].

### 3.8. Effect of Glutamine Supplements on Gut Microbiota in Constipation Groups

In a recent study in which constipated animals were supplemented with glutamine, an increase of intestinal-friendly microbiota from the phyla *Bacteroidetes* and *Actinobacteria* was observed [17]. Indeed, glutamine supplementation can ameliorate constipation and improve intestinal function by regulating the endogenous gut microbiota. In addition to enhancing the energy harvesting capacity by *Firmicutes* spp., the mechanism of glutamine effect on constipation is thought to involve nitrogen balance and protein synthesis in resident bacteria of the small intestine [17]. It is well established that the composition and function of gut microbiota is altered by diet and nutritional status. Accordingly, glutamine supplementation has been proposed as a potential therapeutic approach to alleviating constipation.

### 3.9. Effect of Glutamine on Prevention of Bacterial Translocation

Previous studies have shown some variations in the results regarding the effect of glutamine on bacterial translocation. For instance, Barber et al. [40] reported that glutamine did not prevent bacterial overgrowth or bacterial translocation, whereas other studies showed that glutamine supplementation reduced the intestinal permeability and bacterial translocation due to the ability of the amino acid to preserve the integrity of the intestinal barrier [15,16,41]. Further evidence was reported by dos Santos et al. [18], who demonstrated increased intestinal permeability and bacterial translocation in untreated animals compared to in glutamine-treated animals. Additionally, it has been documented that glutamine and bile may prevent the occurrence of bacterial translocation by decreasing epithelial internalization of enteric bacteria or bacterial adhesion to the intestinal mucosa [42].

### 3.10. Impact of Glutamine Supplement on Gut Microbiota and Immunity

Several studies have shown that the addition of glutamine has a positive effect on the immune response [15,16,18,43]. These in vivo studies and others [44,45,46] were able to demonstrate that glutamine supplementation decreased bacterial colonization and promoted the activation of innate and adaptive immunity. Similar results were reported by Xu et al. [47], who examined the effects of hypobaric hypoxia on gut microbiota composition and found that glutamine significantly alleviated hypobaric hypoxia-induced damage to main organs including the intestine, and increased serum superoxide dismutase (1.14 ± 0.03 vs 0.88 ± 0.04, *p*-value < 0.05) and malondialdehyde (8.35 ± 1.60, *p*-value < 0.01) levels. Furthermore, in another study, glutamine supplementation increased secretory immunoglobulin A (SIgA) concentrations in the jejunum and ileum, and the number of immunoglobulin A+ (IgA^+^) plasma cells in the ileum in mice [48].

### 3.11. Relationship between Glutamine-Enriched Enteral Nutrition and Cytokine Profiles in the Neonatal Period

In a study conducted by Van Zwol et al. [49], it has been observed that, allergic infants had lower counts of bifidobacteria than nonallergic infants. The authors did, however, report significant increases in prevalence of bifidobacteria between the neonatal period and the first year of life in very-low-birth-weight (VLBW) infants. Nevertheless, the study showed that the beneficial effect of receiving enteral preterm formula supplemented with glutamine on infectious morbidity in the neonatal period and allergic diseases was not related to a direct effect on the intestinal microbiota associated with allergy at age 1. Furthermore, Van Zwol et al. [50] indicated that glutamine-enriched enteral nutrition did not influence cytokine profiles in VLBW infants during the neonatal period and the first year of life. More specifically, the authors showed that the beneficial effects of glutamine enteral nutrition on the incidence of serious neonatal infections and atopic dermatitis during the first year of life were not associated with the changes in T helper 1 (Th1) and T helper 2 (Th2) cytokine profiles.

### 3.12. Glutamine Effect on Amino Acids Utilization and Metabolism

In an in vivo study carried out by Dai et al. [51], using a pig model, glutamine administration showed a reduction in the net utilization of asparagine, lysine, leucine, valine, ornithine, and serine by luminal bacteria. Moreover, the authors of the study suggested that extracellular concentrations of glutamine might initiate the signaling pathways related to the metabolism of amino acids in bacterial species of the small intestine.

## 4. Discussion

This review considered the evidence of the glutamine-mediated crosstalk between intestinal microbes and the immune system as well as the effect of glutamine supplementation on intestinal health. Intestinal microbes are known to largely influence the effectiveness of mucosal and systemic immune responses in host mammalian systems [52]. Moreover, amino acid metabolism plays a vital role in the survival and proliferation of the gut microbes [5]. Glutamine, which is the most abundant amino acid in the body and comprises more than 50% of the body’s free amino acid pool, occupies an important position among nutritional metabolism and mammalian host health [53]. Dietary glutamine has received an enormous amount of attention owing to its versatile nature and its many functions in the body. In addition to its ability to stimulate intestinal mucosal hyperplasia [54], glutamine can also initiate the signaling pathways related to the metabolism of nitrogenous compounds in bacteria as demonstrated previously in mammalian cells [51].

Previous studies have demonstrated the importance of glutamine during periods of major physiologic stress [11,27,55]. Glutamine supplementation in patents with serious illness increases levels of immunoglobulin A (IgA) and decreases bacterial translocation [55]. Further, it has been suggested that SIgA promotes aggregation of bacteria in the intestinal lumen, which prevents its adhesion in the epithelial surface, thereby avoiding bacterial translocation. Indeed, results from a study of Ding and Li [53] showed that the levels of mucosal IgA from glutamine treated rats were significantly higher than those in the control and total parenteral nutrition groups. The authors concluded that glutamine supplementation minimized changes in intestinal permeability and bacteria translocation.

Glutamine starvation was initially highlighted by nutritionists, who noticed that plasma glutamine levels reduce significantly during severe injury [56]. Such a concept was further substantiated with several other pathological conditions, which were often associated with hypercatabolic states (e.g., injury, trauma, and sepsis), to compensate the loss of circulating glutamine [57]. For instance, muscle protein catabolism that occurs in cachexia patients is used to maintain plasma glutamine concentrations at a lower level during periods of starvation [58]. As such, glutamine supplementation was reported to reduce muscle breakdown in tumor-bearing animals and protect immune and gut-barrier function in patients with advanced cancer receiving chemo- and radiotherapy [59,60].

Furthermore, studies have suggested that patients receiving chemotherapy and radiotherapy, both of which cause intestinal mucosal damage such as stomatitis and mucositis, may also benefit from oral glutamine [61,62,63]. However, oral and parenteral glutamine supplementation has produced inconsistent results concerning the prevention of chemotherapy-induced oral mucositis. For example, Ward et al. [64] failed to demonstrate any beneficial effect of glutamine in preventing oral mucositis in children undergoing chemotherapy.

The effects of glutamine supplementation on obesity were also studied. In a recent study by Abboud et al. [65], glutamine supplementation was demonstrated to induce a reduction in waist circumference and serum lipopolysaccharide concentration in overweight volunteers. The authors also reported a reduction in insulin action and glucose uptake in fat and adipose mass of rats fed with a high-fat diet. Further, the association of glutamine supplementation and shifts in gut microbiota composition in obese and overweight individuals was evident [3].

## 5. Materials and Methods

The present systematic review was performed according to the steps developed by Egger et al. [66] as follows:

1. Configuration of a working group: three operators skilled in clinical nutrition with one acting as a methodological operator and two participating as clinical operators;
2. Formulation of the revision question on the basis of considerations made in the abstract: “The role of glutamine in the complex interaction between gut microbiota and disease”;
3. Identification of relevant studies: a research strategy was planned on PubMed and Scopus as follows:
  - definition of the keywords allowing for the definition of the interest field of the documents to be searched, grouped in inverted commas (“…”), and used separately or in combination;
  - use of the Boolean AND operator, which allows for the establishment of logical relationships among concepts;
  - research modalities: advanced search;
  - limits: papers published until June 2019; humans, animals, in vivo, and in vitro studies; languages: English;
  - manual search performed by senior researchers experienced in clinical nutrition through the revision of reviews and research articles on glutamine and gut published in qualified journals of the Index Medicus.

## 6. Conclusions

This review has highlighted the potential applications of glutamine supplementation. Nevertheless, data on the precise mechanisms by which glutamine exerts its effects are still limited. Therefore, additional clinical trials and evaluation studies are required to better define glutamine’s purported effects in order to maximize its use for nutritional support.
